# Study of Fibrinogen Albumin Ratio in Type 2 Diabetic Patients and Its Correlation With Diabetic Peripheral Neuropathy

**DOI:** 10.7759/cureus.74977

**Published:** 2024-12-02

**Authors:** Mitali B Rathod, Anuka Teja Reddy, Bhavya Nagaraju, Chanthu Jr, Dheepika Arumugaperumal, Maria Sneha Ashwini

**Affiliations:** 1 General Medicine, NAMO Medical College and Research Centre, Silvassa, IND; 2 Department of General Medicine, International School of Medicine, Bishkek, KGZ; 3 Department of General Medicine, KAP Viswanatham Government Medical College, Tiruchirappalli, IND; 4 Department of General Medicine, Dhanalakshmi Srinivasan Medical College and Hospitals, Perambalur, IND; 5 Department of General Medicine, Father Muller Medical College, Bangalore, IND

**Keywords:** correlation, diabetic kidney disease, diabetic peripheral neuropathy, fibrinogen, fibrinogen to albumin ratio, inflammation, nerve conduction studies, type 2 diabetes mellitus

## Abstract

Background: Type 2 diabetes mellitus (T2DM) is a chronic metabolic disorder that imposes significant complications, including diabetic peripheral neuropathy (DPN). DPN is characterized by marked inflammation, and the fibrinogen-to-albumin ratio (FAR) is one of the new markers for systemic inflammation, it has been used in various diabetic micro- and macro-vascular complications. The present study investigates the association between FAR and nerve conduction abnormalities in T2DM patients with DPN.

Methods: This was a cross-sectional study conducted on 200 T2DM patients, and 110 (55%) were diagnosed with DPN. Patients were divided into DPN and non-DPN groups. The patient's demographics and the biochemical variables fibrinogen, albumin, and FAR were evaluated and compared among the DPN and non-DPN groups. Nerve conduction studies (NCS) were performed to evaluate motor and sensory nerve functions. Patients were further stratified into FAR tertiles (low, moderate, and high FAR) for subgroup analysis.

Results: The fibrinogen levels were significantly higher (375.65 ± 35.43 mg/dL vs. 342.87 ± 42.12 mg/dL; p<0.001) and albumin levels were lower (3.12 ± 0.41 g/dL vs. 4.07 ± 0.57 g/dL; p<0.001) in DPN as compared to non-DPN patients. Meanwhile, FAR was higher in DPN as compared to non-DPN patients (120.40 ± 26.32 vs. 84.24 ± 12.87; p<0.001). Further, there was a significant negative correlation for conduction velocities and amplitudes and a positive correlation for latencies with FAR across all nerves tested (p<0.05). In addition, high FAR tertile patients showed lower conduction velocities (peroneal nerve: 35.26 ± 5.82 m/s vs. 42.53 ± 5.34 m/s; p<0.001), reduced amplitudes, and prolonged latencies when compared to moderate and low FAR tertile patients (p<0.05).

Conclusion: Nerve conduction abnormalities in T2DM patients with DPN showed significant association with FAR. Thus, FAR serves as a simple and effective biochemical marker for the assessment of DPN severity.

## Introduction

Type 2 diabetes mellitus (T2DM) is a chronic metabolic disorder characterized by insulin resistance, and it imposes significant morbidity and mortality. According to the international diabetes federation (IDF), type 2 diabetes affects approximately 463 million people globally (9.3% of the global adult population), and the estimate is expected to increase to 700 million by 2045 [1]. The prevalence of T2DM varies by region, with the highest rates reported in regions such as Southeast Asia, the Middle East, and North Africa. India has one of the highest burdens of T2DM, with around 77 million people affected, representing a prevalence rate of about 8-10% in the adult population. India is expected to see a steep increase in cases, possibly surpassing 100 million by 2045 [1].

Diabetic peripheral neuropathy (DPN) is a macrovascular complication of T2DM, and it affects the physical performance of diabetic individuals. DPN also exerts painful events such as neuropathic pain and diabetic foot diseases, including diabetic foot ulcers, and further elevates the risk of limb amputations and mortality [2]. Early detection, diagnosis, and treatment of DPN are important for managing the condition and preventing its progression. It is also essential to control blood glucose levels and manage other risk factors to prevent the development of DPN in patients with T2DM [3].

Among various inflammatory markers, fibrinogen has gained attention for its potential role in diabetic complications. Fibrinogen, an acute-phase protein, is not only a marker of inflammation but also plays a role in blood coagulation, thus contributing to vascular complications [4]. Elevated fibrinogen levels have been associated with both T2DM and its complications, particularly those of a vascular nature [5]. On the other hand, albumin, a negative acute-phase protein, often decreases in chronic inflammatory states [6]. Thus, the fibrinogen-to-albumin ratio (FAR) has emerged as a potential biomarker reflecting both inflammatory and nutritional status, including cardiovascular disease and cancer [7,8].

Recent studies have suggested that FAR may correlate with the severity of diabetic complications, particularly those involving vascular pathology DPN [9]. Given that DPN shares common pathogenic pathways with other diabetic macrovascular complications [10], FAR could be a valuable marker in predicting the risk or progression of neuropathy in diabetic patients. However, there is limited data specifically addressing the relationship between FAR and DPN in T2DM patients.

This study aims to evaluate the fibrinogen-albumin ratio in T2DM patients and explore its correlation with diabetic peripheral neuropathy. Understanding the association between FAR and DPN could offer insights into the underlying inflammatory mechanisms and potentially aid in identifying patients at higher risk for developing neuropathy, thus improving early intervention strategies and patient outcomes.

## Materials and methods

This cross-sectional study was conducted at the Department of General Medicine, NAMO Medical College and Research Centre, Silvassa, India, for one year, from August 2023 to August 2024. The study included 200 patients diagnosed with type 2 diabetes mellitus (T2DM) based on the American Diabetes Association (ADA) criteria. The study was approved by the institutional ethical committee of Namo Medical Education & Research Institute, Silvassa, India, with a protocol number (IEC/2023/127/225).

Inclusion criteria

Patients aged 30 to 70 years with a confirmed diagnosis of T2DM for at least 5 years were eligible to participate. Patients were screened for classic neuropathy symptoms, such as numbness, tingling, or burning sensations, loss of sensation, muscle weakness in the limbs, and sharp and stabbing pain.

Exclusion criteria

Patients with other causes of neuropathy (e.g., alcohol-induced or chemotherapy-related neuropathy), recent infections or acute inflammation, chronic liver disease, renal impairment (eGFR < 30 mL/min/1.73 m²), or any recent surgeries were excluded from the study. Patients with a history of conditions that could confound nerve function assessment, such as spinal cord disorders, were also excluded from the study.

Data collection

Eligible participants were recruited from the diabetes outpatient clinic. Written informed consent was obtained from each participant prior to enrolment. Demographic data, clinical history, and biochemical data were collected, including age, gender, duration of diabetes, and use of medications such as insulin and antihypertensive agents.

Assessment of diabetic peripheral neuropathy (DPN)

Diabetic peripheral neuropathy was assessed using the Michigan Neuropathy Screening Instrument (MNSI) and nerve conduction studies (NCS). The MNSI includes a history questionnaire and a physical examination, with scores above a specific threshold indicating the presence of neuropathy. Nerve conduction studies (NCS) were conducted on the nondominant hand side, specifically targeting the median, tibial, sural, and medial plantar nerves. The tests were performed using a Neuropack S1 device (Nihon Kohden, Tokyo, Japan), with the bandpass filter set between 5 Hz and 5 kHz. Participants were categorized into two groups: those with DPN and those without DPN.

Biochemical analysis

Blood samples were drawn after an overnight fast of 8-10 hours. Glycosylated hemoglobin (HbA1c) was measured using high-pressure liquid chromatography. Serum fibrinogen levels were measured using the Clauss method, while serum albumin levels were measured by a colorimetric assay. The fibrinogen-to-albumin ratio (FAR) was calculated as: 

FAR= Fibrinogen (mg/dL)/albumin (g/dL). The FAR tertiles were done as follows, Low risk FAR: < 75, Moderate risk FAR: 75-90, and High risk FAR: > 90 [10].

Statistical analysis

Statistical analysis was performed in IBM Corp. Released 2020. IBM SPSS Statistics for Windows, Version 27.0. Armonk, NY: IBM Corp. Descriptive statistics were used to summarize the demographic and clinical characteristics of the study population. The FAR values were compared between patients with and without DPN using the independent t-test or Mann-Whitney U test as appropriate. The correlation between FAR and the severity of DPN (as assessed by NCS) was evaluated using Pearson’s correlation coefficient analysis. Multivariable logistic regression analysis was conducted to adjust for potential confounding variables, including age, gender, duration of diabetes, and HbA1c levels. The comparison of variables across the FAR tertiles was done by one-way ANOVA. A p-value < 0.05 was considered statistically significant.

## Results

The study was conducted on 200 diabetic patients, and the prevalence of DPN was 110 (55%) among the study population. The patients were divided into DPN and non-DPN groups. In this study, there was a significant difference in the age (57.1 ± 9.8 vs. 53.0 ± 10.4; p=0.04), HbA1c (10.65 ± 2.32 vs. 7.42 ± 2.76; p=0.02) and the incidence of hypertension among the DPN and non-DPN groups. The results are shown in Table 1.

**Table 1:** Comparison of demographics and clinical characteristics of DPN and Non-DPN T2DM patients DPN: Diabetic peripheral neuropathy; a: Unpaired students t-test; b: Chi square test; * indicates statistically significant (p<0.05), NS: Non-significant

Variables	Total (N=200)	DPN Group (n=110)	Non-DPN Group (n=90)	p-value
Age (years)	55.3 ± 10.2	57.1 ± 9.8	53.0 ± 10.4	0.04^a*^
Gender (Male)	110 (55%)	58 (52.7%)	52 (57.8%)	0.48^ bNS^
Duration of Diabetes (years)	8.6 ± 3.2	9.12 ± 3.34	8.32 ± 3.16	0.07^ aNS^
HbA1c (%)	9.03 ± 1.2	10.65 ± 2.32	7.42 ± 2.76	0.02^ a*^
Body Mass Index (BMI) (kg/m²)	27.54 ± 4.3	28.32 ± 4.0	26.8 ± 4.87	0.07^ aNS^
Hypertension (%)	120 (60%)	75 (68.2%)	45 (50%)	0.01^ a*^

**Figure 1:**
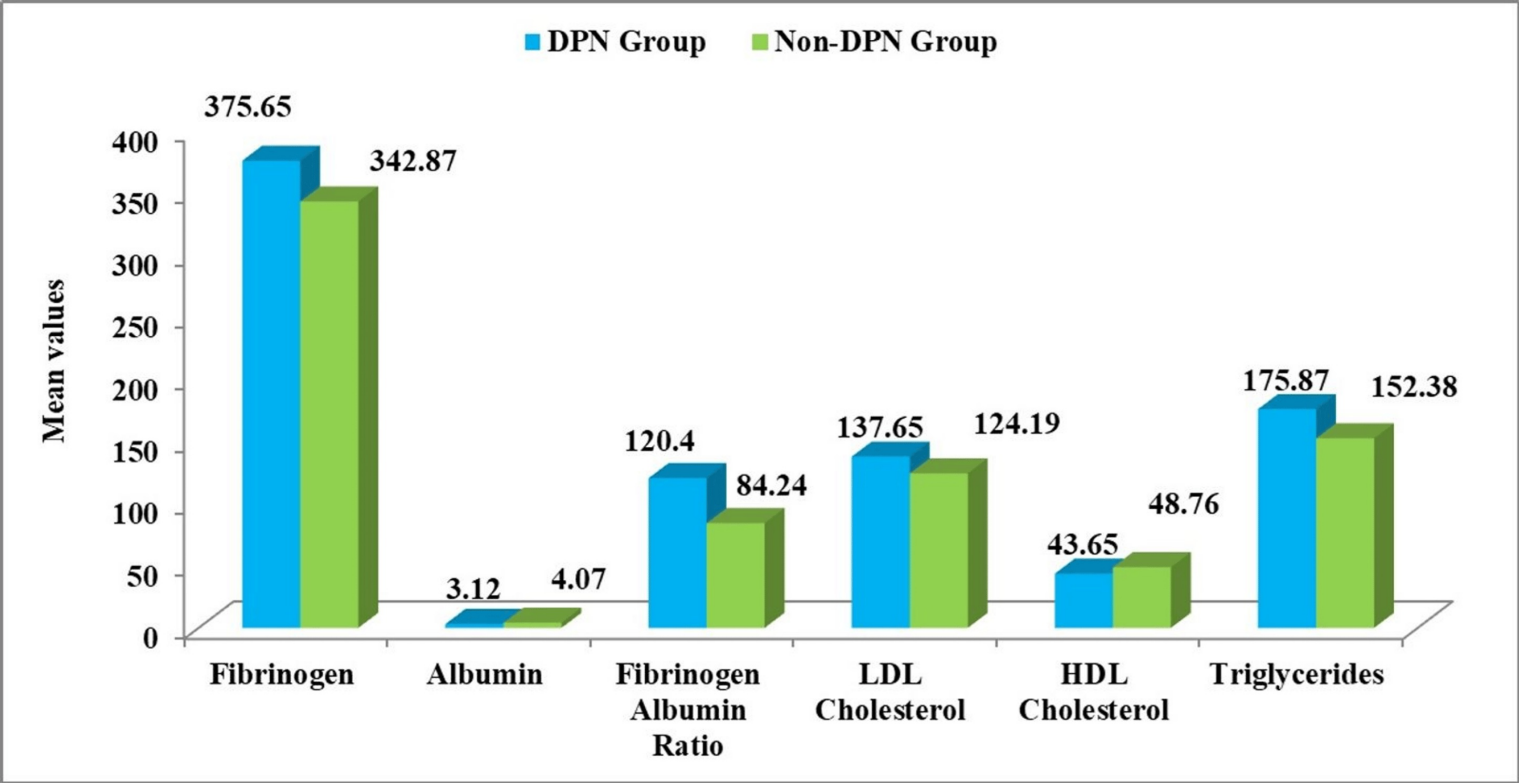
Comparison of fibrinogen, albumin, FAR, and other biochemical parameters among the DPN and non-DPN T2DM patients DPN: Diabetic peripheral neuropathy; LDL: Low density lipoprotein; HDL: High density lipoprotein

In this study, the biochemical parameters were compared among the DPN and the non-DPN cases. The fibrinogen levels were higher in DPN when compared to the non-DPN T2DM patients, and it was significant (375.65 ± 35.43 mg/dL vs. 342.87 ± 42.12 mg/dL; p<0.001). Meanwhile, the albumin level was lower in DPN when compared to the non-DPN T2DM patients, and it was significant (3.12 ± 0.41 g/dL vs. 4.07 ± 0.57 g/dL; p<0.001). In addition, the fibrinogen albumin ratio was significantly higher in DPN when compared to non-DPN T2DM patients (120.40 ± 26.32 vs. 84.24 ± 12.87; p<0.001). The results are shown in Figure 1.

In the present study, across all nerves tested, the DPN group consistently showed significantly reduced conduction velocities compared to the non-DPN group (p<0.05), indicating delayed nerve conduction in patients with DPN. Both motor and sensory amplitudes were significantly (p<0.05) lower in the DPN Group across all nerves, suggesting reduced signal strength in diabetic patients with neuropathy. The DPN group demonstrated longer latencies compared to the non-DPN group in most nerves tested, reflecting slower neural response times, and it was found to be significant (p<0.05). The results are shown in Table 2.

**Table 2:** Comparison of Nerve Conduction Study Parameters among the DPN and non-DPN T2DM patients DPN: Diabetic peripheral neuropathy. The data is shown as mean±SD. Unpaired students t-test; * indicates statistically significant (p<0.05)

Nerve Studied	Parameter	DPN Group (n=110)	Non-DPN Group (n=90)	p-value
Peroneal Nerve	Conduction Velocity (m/s)	35.27 ± 5.87	42.12 ± 6.24	<0.001^*^
	Amplitude (mV)	2.31 ± 0.65	3.15 ± 0.72	<0.001*
	Latency (ms)	5.12±0.8	4.54±0.7	0.02*
Tibial Nerve	Conduction Velocity (m/s)	36.81±5.52	43.28±5.87	<0.001*
	Amplitude (mV)	2.69±0.52	3.35±0.81	<0.001*
	Latency (ms)	4.84±0.92	4.17±0.75	0.01*
Sural Nerve (Sensory)	Conduction Velocity (m/s)	39.16±7.54	45.73±8.32	<0.001*
	Amplitude (µV)	5.14±1.65	6.39±2.54	<0.001*
	Latency (ms)	3.32±0.56	2.84±0.43	0.02*
Median Nerve (Motor)	Conduction Velocity (m/s)	42.24±7.31	46.58±8.54	0.01*
	Amplitude (mV)	3.18±0.72	3.73±0.54	0.03*
	Latency (ms)	4.64±0.65	4.14±0.43	0.02*
Median Nerve (Sensory)	Conduction Velocity (m/s)	44.52 ± 6.24	49.29±5.34	<0.001*
	Amplitude (µV)	4.24±1.45	5.48±2.04	<0.001*
	Latency (ms)	3.24±0.65	2.72±0.24	0.03*
Ulnar Nerve (Motor)	Conduction Velocity (m/s)	43.52±7.25	47.83±6.87	0.01
	Amplitude (mV)	2.76±0.52	3.59±0.87	0.02*
	Latency (ms)	5.14±1.12	4.29±0.98	0.03*
Ulnar Nerve (Sensory)	Conduction Velocity (m/s)	45.38±7.12	50.52±9.43	<0.001*
	Amplitude (µV)	4.76±1.28	5.62±1.82	<0.001*
	Latency (ms)	3.18±0.42	2.67±0.34	0.02*

In this study, the conduction velocities for peroneal nerve (r=-0.45; p<0.001), tibial nerve (r= -0.48; p<0.001), sensory sural nerve (r= -0.53; p<0.001), motor median nerve (r= -0.46; p<0.001), sensory median nerve (r=-0.50; p<0.001), motor ulnar nerve (r= -0.44; p<0.001), sensory ulnar nerve (r=-0.48; p<0.001) showed significant negative correlation with FAR in T2DM patients with DPN. Likewise, the signal amplitude for peroneal nerve (r=-0.38; p=0.002), tibial nerve (r= -0.35; p=0.005), sensory Sural nerve (r= -0.41; p=0.001), motor median nerve (r= -0.34; p=0.006), sensory median nerve (r=-0.50; p<0.001), motor ulnar nerve (r= -0.44; p<0.001), sensory ulnar nerve (r=-0.36; p<0.001) showed significant negative correlation with FAR in T2DM patients with DPN. Meanwhile, the latency impulses for peroneal nerve (r=0.32; p=0.01), tibial nerve (r= 0.28; p=0.03), sensory Sural nerve (r= 0.30; p=0.02), motor median nerve (r=0.25; p=0.03), sensory median nerve (r=0.31; p=0.01), motor ulnar nerve (r= 0.30; p=0.02), sensory ulnar nerve (r=0.33; p=0.01) showed significant positive correlation with FAR in T2DM patients with DPN. The results are shown in Table 3.

**Table 3:** Correlation between nerve conduction studies and FAR in T2DM patients with DPN Pearson’s correlation analysis; * indicates statistically significant (p<0.05)

Nerve Studied	Parameter	Pearson’s correlation (r)	p-value
Peroneal Nerve	Conduction Velocity (m/s)	-0.45	<0.001*
	Amplitude (mV)	-0.38	0.002*
	Latency (ms)	0.32	0.01*
Tibial Nerve	Conduction Velocity (m/s)	-0.48	<0.001*
	Amplitude (mV)	-0.35	0.005*
	Latency (ms)	0.28	0.03*
Sural Nerve (Sensory)	Conduction Velocity (m/s)	-0.53	<0.001*
	Amplitude (µV)	-0.41	0.001*
	Latency (ms)	0.30	0.02*
Median Nerve (Motor)	Conduction Velocity (m/s)	-0.46	<0.001*
	Amplitude (mV)	-0.34	0.006*
	Latency (ms)	0.25	0.03*
Median Nerve (Sensory)	Conduction Velocity (m/s)	-0.50	<0.001*
	Amplitude (µV)	-0.42	0.002*
	Latency (ms)	0.31	0.01*
Ulnar Nerve (Motor)	Conduction Velocity (m/s)	-0.44	<0.001*
	Amplitude (mV)	-0.36	0.004*
	Latency (ms)	0.30	0.02*
Ulnar Nerve (Sensory)	Conduction Velocity (m/s)	-0.48	<0.001*
	Amplitude (µV)	-0.40	0.003*
	Latency (ms)	0.33	0.01*

In the present study, the fibrinogen albumin ratio (FAR) levels were divided into the following tertiles: low FAR (<75), moderate FAR (75-90), and high FAR (>90), respectively. There was a significant difference in the age (p=0.03), duration of diabetes (p=0.04), HbA1c (p=0.01), and DPN prevalence (p=0.02) across the FAR tertiles. Further, the fibrinogen levels (397.54 ± 35.64 mg/dL vs. 360.65 ± 32.82 mg/dL and 320.43 ± 28.25 mg/dL; p<0.001) were higher in patients with high FAR tertiles when compared to moderate and low FAR tertiles, and it was significant. Meanwhile, the albumin level was lower (3.09 ± 0.48 g/dL vs. 3.93 ± 0.45 g/dL and 4.12± 0.57g/dL) in patients with high FAR tertiles when compared to moderate and low FAR tertiles, and it was significant. The results are shown in Table 4.

**Table 4:** Comparison of demographics and biochemical parameters between the FAR tertiles The data is shown as mean±SD. One-way ANOVA. * indicates statistically significant (p<0.05); NS: Non-significant

Parameter	Low FAR (<75) (n=70)	Moderate FAR (75–90) (n=65)	High FAR (>90) (n=65)	p-value
Age (years)	52.32 ± 9.24	56.17 ± 10.29	58.24 ± 11.16	0.03*
Duration of Diabetes (years)	7.54 ± 3.25	8.73 ± 3.44	9.51 ± 3.26	0.04*
Male (%)	40 (57.14%)	35 (53.84%)	35 (53.81%)	0.92^NS^
HbA1c (%)	7.4 ± 1.0	7.8 ± 1.2	8.2 ± 1.4	0.01*
DPN Prevalence (%)	30 (42.85%)	38 (58.46%)	42 (64.61%)	0.02*
Fibrinogen (mg/dL)	320.43 ± 28.25	360.65 ± 32.82	397.54 ± 35.64	<0.001*
Albumin (g/dL)	4.12± 0.57	3.93 ± 0.45	3.09 ± 0.48	<0.001*

In the present study, the conduction velocity and amplitude were significantly lower, and the latency was higher for the peroneal nerve, tibial nerve, sensory sural nerve, motor median nerve, sensory median nerve, and motor ulnar nerve in high FAR groups as compared to moderate and low FAR groups, and it was significant (p<0. 05). The results were shown in Table 5.

**Table 5:** Comparison of nerve conduction studies between the FAR tertiles The data is shown as mean±SD. One-way ANOVA. * indicates statistically significant (p<0.05); NS-Non significant

Parameter	Low FAR (<75) (n=70)	Moderate FAR (75–90) (n=65)	High FAR (>90) (n=65)	p-value
Peroneal Nerve				
Conduction Velocity (m/s)	42.53 ± 5.34	38.87 ± 5.11	35.26 ± 5.82	<0.001*
Amplitude (mV)	3.56 ± 0.81	2.65 ± 0.72	2.32 ± 0.64	<0.001*
Latency (ms)	4.27 ± 0.67	4.76 ± 0.78	5.05 ± 0.82	0.02*
Tibial Nerve				
Conduction Velocity (m/s)	43.23 ± 5.61	39.78 ± 4.28	36.87 ± 5.45	<0.001*
Amplitude (mV)	3.29 ± 0.71	2.93 ± 0.62	2.63 ± 0.65	<0.001*
Latency (ms)	4.21 ± 0.65	4.52 ± 0.51	4.83 ± 0.72	0.01*
Sural Nerve (Sensory)				
Conduction Velocity (m/s)	45.67 ± 6.11	42.48 ± 5.92	39.12 ± 6.04	<0.001*
Amplitude (µV)	6.31 ± 1.13	5.55 ± 1.31	5.02 ± 1.21	<0.001*
Latency (ms)	2.73 ± 0.43	3.03 ± 0.54	3.32 ± 0.58	0.02*
Median Nerve (Motor)				
Conduction Velocity (m/s)	46.58± 5.17	43.52 ± 5.34	42.25 ± 5.42	0.01*
Amplitude (mV)	3.73 ± 0.64	3.46 ± 0.67	3.12 ± 0.71	0.03*
Latency (ms)	4.12 ± 0.42	4.43 ± 0.51	4.68 ± 0.54	0.02*
Median Nerve (Sensory)				
Conduction Velocity (m/s)	49.23 ± 6.03	46.12 ± 5.82	44.53 ± 6.14	<0.001*
Amplitude (µV)	5.46 ± 1.13	4.82 ± 1.22	4.26 ± 1.01	<0.001*
Latency (ms)	2.72 ± 0.41	3.03 ± 0.54	3.28 ± 0.51	0.03*
Ulnar Nerve (Motor)				
Conduction Velocity (m/s)	50.53 ± 6.13	46.72 ± 5.92	45.35 ± 6.18	<0.001*
Amplitude (µV)	5.68 ± 1.21	4.98 ± 1.35	4.18 ± 1.32	<0.001*
Latency (ms)	2.66 ± 0.43	3.02 ± 0.64	3.16 ± 0.72	0.02*

## Discussion

Diabetic peripheral neuropathy (DPN) is one of the most common and debilitating complications of type 2 diabetes mellitus (T2DM), characterized by sensory and motor nerve dysfunction. Chronic hyperglycemia induces oxidative stress, inflammation, and vascular damage, which play pivotal roles in the pathogenesis of DPN. The fibrinogen-to-albumin ratio (FAR) is a sensitive indicator of the body’s inflammatory response. Combining serum albumin and fibrinogen levels, FAR provides a reliable measure of systemic inflammation and is valuable in predicting outcomes in various conditions, including cardiovascular diseases and cancer [11,12]. This study aimed to investigate the relationship between FAR and nerve conduction parameters in T2DM patients, with stratification into FAR tertiles to assess its utility as a marker for DPN severity. The results highlight significant trends linking higher FAR levels to worsening nerve function.

In the present study, the prevalence of DPN among T2DM patients is estimated to be 55%. In India, the prevalence of DPN ranges between 18.8 and 61.9% [13, 14]. The present study results showed that the HbA1c levels (10.65 ± 2.32 vs. 7.42 ± 2.76; p = 0.02) and the prevalence of hypertension (68.2% vs. 50%) were significantly higher in DPN T2DM patients as compared to non-DPN T2DM patients. In a study done by Bansal et al., the incidence of hypertension was higher in DPN as compared to non-DPN diabetic patients (38.7% vs. 31.9%; p = 0.03) [15]. In a study done by Lu et al., the mean HbA1c level was higher in DPN cases compared to non-DPN cases and was significant (8.70 vs 7.97; p<0.001) [16]. In the present study, the fibrinogen levels were higher in DPN when compared to the non-DPN T2DM patients, and it was significant (375.65 ± 35.43 mg/dL vs. 342.87 ± 42.12 mg/dL; p<0.001). Meanwhile, the albumin level was lower in DPN compared to the non-DPN T2DM patients, and it was significant (3.12 ± 0.41 g/dL vs. 4.07 ± 0.57 g/dL; p<0.001). This finding reveals the role of fibrinogen as a marker of systemic inflammation, which contributes to microvascular damage and neural injury in diabetes. Elevated fibrinogen levels are known to enhance blood viscosity, promote clot formation, and impair microcirculation, exacerbating nerve ischemia and oxidative stress-key mechanisms in the pathogenesis of DPN [17]. Conversely, albumin levels were significantly lower in patients with DPN than in those without DPN. Reduced albumin levels are indicative of poor nutritional status and systemic inflammation, both of which negatively affect neural repair mechanisms and contribute to worsening neuropathy [18]. Hypoalbuminemia also reflects compromised antioxidant defenses, further amplifying oxidative stress and neural damage in DPN. Likewise, in a study done by Ban et al., there was a significant increase in fibrinogen levels (2.76 vs. 2.53 g/L; p<0.001) and a decrease in albumin level (41.4 vs. 42.82 g/L) in DPN as compared to the non-DPN group [9]. Likewise, in another study done by Zhao et al., the fibrinogen level (3.89 vs. 3.00 g/L; p<0.001) was increased and the albumin level (39.49 vs. 42.29g/l; p<0.001) was decreased in patients with diabetic cardiac autonomic neuropathy (DCAN) and without DCAN [19].

In the present study, the fibrinogen albumin ratio (FAR) was significantly higher in DPN when compared to non-DPN T2DM patients (120.40 ± 26.32 vs. 84.24 ± 12.87; p<0.001). Likewise, in a study done by Ban et al., the FAR level was significantly increased in DPN as compared to non-DPN cases (7.39 vs. 5.59; p<0.001) [9]. In another study done by Chen et al., the FAR level was elevated in diabetic retinopathy as compared to non-diabetic retinopathy cases (6.83 vs. 5.86; p<0.001) [20].

The present study demonstrated that DPN is associated with significant impairments in nerve conduction parameters across all nerves tested. The DPN group exhibited consistently reduced conduction velocities compared to the non-DPN group, highlighting delayed neural signal transmission. This reduction reflects the impact of demyelination and axonal damage, both of which are characteristic features of DPN caused by chronic hyperglycemia, oxidative stress, and inflammation. Both motor and sensory nerve amplitudes were significantly lower in the DPN group, indicating reduced signal strength [21]. This finding suggests a loss of functional nerve fibers and impaired neural integrity, contributing to the clinical manifestations of neuropathy, such as sensory deficits and muscle weakness. Additionally, the longer latencies observed in the DPN group indicate slower neural response times, reflecting disruptions in the conduction pathways. These changes in latency, coupled with reduced velocities and amplitudes, provide robust electrophysiological evidence of nerve dysfunction in DPN patients [22]. The statistical significance (p<0.05) across these parameters further underscores the consistent and profound impact of DPN on peripheral nerve function. Likewise, in a study done by Ban et al., DPN patients showed slower sensory and motor conduction velocities in various nerves, including the median nerve, superficial peroneal nerve, sural nerve, and ulnar nerve, when compared to patients without DPN, and it was significant (p<0.05) [9]. In another study done by Agarwal et al., the motor and sensory NCS in median, ulnar, common peroneal, and posterior tibial nerves were slower in DPN as compared to non-DPN cases (p<0.001) [23]. In the same study, the motor and sensory latencies in median, ulnar, and sural nerves were higher in DPN as compared to non-DPN patients (p< 0.001) [23].

In this study, the conduction velocities and signal amplitude showed negative correlation, and latency impulses showed positive correlation with FAR for the peroneal nerve, tibial nerve, sensory sural nerve, motor median nerve, sensory median nerve, motor ulnar nerve, and sensory ulnar nerve, and it was significant (p<0.05). This indicates that higher FAR values, representing increased inflammation, are associated with slower nerve signal transmission and reduced neural signal strength. Conversely, latency impulses exhibited a positive correlation with FAR, suggesting that elevated FAR levels are linked to delayed nerve response times [24]. Likewise, in a study done by Ban et al., there is a significant negative correlation between FAR levels and DPN nerve conduction velocity [9].

In the present study, the FAR levels were categorized into three tertiles, such as low, moderate, and high. The HbA1 (p=0.01), DPN prevalence (p=0.02), fibrinogen (p<0.001), and albumin (p<0.001) were significantly higher in the FAR high tertile as compared to the moderate and low tertile. Likewise, in a study done by Ban et al., the HbA1c (8.7% vs. 7.9%; p<0.001) and fibrinogen (2.99 vs. 2.35g/L; p<0.001) were significantly higher, and the albumin level (40.5 vs. 42.8g/L; p<0.001) was significantly lower in the high FAR group as compared to the low FAR group [9]. In the present study, the conduction velocity and amplitude were significantly lower, and the latency was higher for various nerves in the high FAR tertile as compared to the moderate and low FAR tertile, and it was significant (p<0. 05). Likewise, in a study done by Ban et al., there was a slower conduction velocity and amplitude in the high FAR group as compared to the low FAR group, and it was significant (p<0.05) [9].

The main limitations of the study were single-center research and a relatively small sample size. The study did not consider other potential confounders, such as detailed lipid profiles, advanced glycation end products, or genetic predispositions, which might influence nerve conduction and inflammation.

## Conclusions

The present study demonstrated that the fibrinogen-to-albumin ratio (FAR) is significantly associated with nerve conduction abnormalities in type 2 diabetes mellitus patients with DPN. Higher FAR levels were linked to slower conduction velocities, lower signal amplitudes, and prolonged latencies across both motor and sensory nerves, indicating its role as a marker for neural dysfunction. These findings underscore the importance of FAR as a simple and reliable biomarker for assessing systemic inflammation and nutritional status in DPN patients. Future studies with larger, diverse populations and longitudinal designs are warranted to validate these findings and explore the potential of FAR in guiding therapeutic interventions for DPN.
